# Prophylactic antibiotics for miscarriage surgery: A protocol for systematic review and meta-analysis

**DOI:** 10.1097/MD.0000000000020959

**Published:** 2020-07-02

**Authors:** Yu Fu, Ruirui Jin, Xiaoxia Wang, Qingmei Sun, Xiaojuan Lin, Xiaozhuan Wang, Zhongfeng Tang, Xiaoyu Song, Youhong Zhao

**Affiliations:** a Gansu Provincial Maternity and Child-care Hospital; b Gansu Gem Flower Hospital, Lanzhou city, Gansu province, China.

**Keywords:** meta-analysis, miscarriage surgery, prophylactic antibiotics

## Abstract

**Background:**

Infection is a serious potential consequence of surgery to complete a spontaneous abortion. Antibiotic prophylaxis before some operations has been shown to reduce the risk of postoperative infections. However, for miscarriage surgery, evidence is lacking to show effectiveness.

**Methods:**

In this systematic review, the electronic databases of Cochrane Central Register of Controlled Trials, EMBASE, and PUBMED will be searched from inception to May 1, 2020. Randomized controlled trials that assessed the effectiveness and safety of antibiotic prophylaxis for preventing infection for patients undergoing miscarriage surgery will be included. All process of the study selection, data extraction, and methodology evaluation will be carried out by two authors independently. RevMan 5.3 software will be utilized for statistical analysis.

**Results:**

This study will provide a detailed summary of latest evidence related to the effectiveness and safety of antibiotic prophylaxis for preventing infection for patients undergoing miscarriage surgery.

**Conclusion:**

The findings of this study may provide possible guidance for the use of antibiotic prophylaxis for preventing infection for patients undergoing miscarriage surgery.

**Dissemination and ethics:**

Ethical approval is not required in this study, because it will not collect the original data from individual patient. The results are expected to publish through a peer-reviewed journal.

**Systematic review registration:**

PROSPERO CRD CRD42020155643

## 1 Introduction

Globally, 208 million women and adolescents become pregnant each year,^[1]^ but 10 to 20% of pregnancies end in spontaneous abortion.^[2]^ In many of these cases, surgery is needed to remove retained products of conception^[3]^; such surgery is 1 of the most common gynecologic operations performed worldwide and infection is a serious potential consequence of surgery to complete a spontaneous abortion, in particular in low- and middle-income countries.^[4]^ Pelvic infection can result in serious illness and death,^[5]^ as well as long-term consequences from pelvic scarring, including increased rates of ectopic pregnancy and infertility.^[6]^

Antibiotic prophylaxis before some operations has been shown to reduce the risk of postoperative infections. A Cochrane review of 19 randomized, controlled trials of the use of antibiotic prophylaxis before uterine evacuation for induced termination of pregnancy showed that prophylactic antibiotics reduced pelvic infection for this specific indication.^[7]^ However, for miscarriage surgery, evidence is lacking to show effectiveness,^[8]^ with four small, single-center studies showing no significant benefit from prophylactic antibiotics. In addition to small size,^[4,9–11]^ these studies had other methodologic limitations, including inadequate antibiotic dose ^[9]^ and poor adherence to the study protocol.^[4]^

International guidelines regarding antibiotic prophylaxis for surgery for incomplete spontaneous abortion are inconsistent. Some do not recommend antibiotics, reflecting the lack of evidence of efficacy,^[12–14]^ whereas others acknowledge the lack of evidence but still advocate for their use on the basis of extrapolation of findings from other indications.^[15]^

The question of whether to use prophylactic antibiotics is particularly important in low- and middle-income countries. Rates of surgery for incomplete spontaneous abortion are high owing to low uptake of nonsurgical management approaches,^[16]^ a higher incidence of infections after surgery in these countries than in high-income countries,^[17–19]^ and poor access to resources to care for women in whom complications develop.^[20]^ High-quality evidence is needed for rational antimicrobial prescribing.^[21]^

We plan to design a systematic review and meta-analysis to investigate whether, among women and adolescents undergoing surgery for incomplete spontaneous abortion, the use of prophylactic antibiotics before the miscarriage surgery would reduce the risk of pelvic infection.

## 2 Methods

The guidelines for this systematic review were based on preferred reporting items for systematic reviews and meta-analysis protocol (PRISMA) recommendations, and a protocol for this review was published in PROSPERO with the registration number CRD42020155643. Systematic Reviews of Interventions and the PRISM Protocol statement guidelines.^[22]^

### 2.1 Literature search strategy

**Figure 1:**
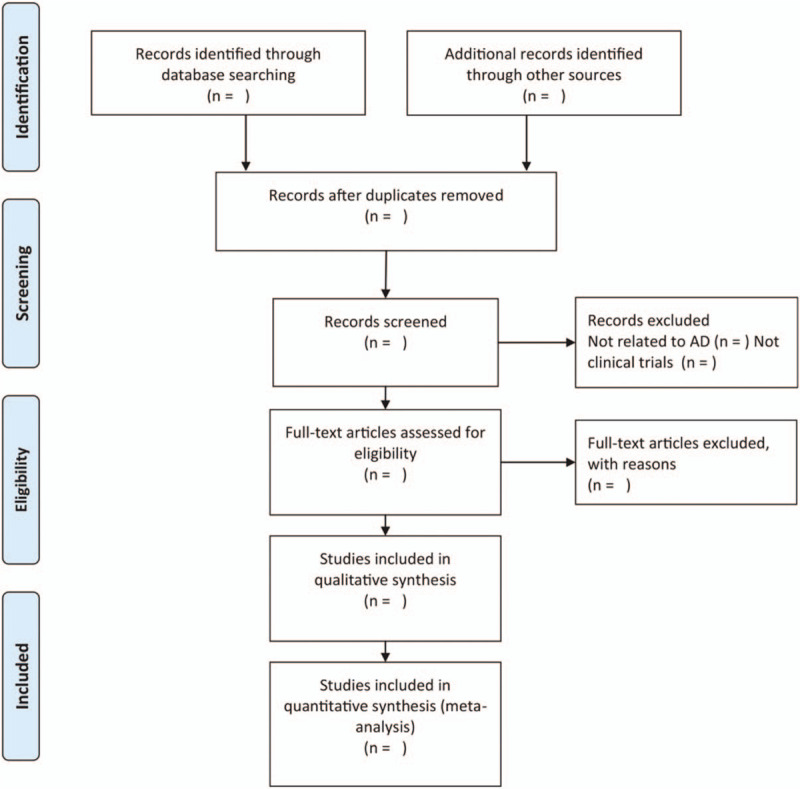
Flow diagram of literature screening.

An electronic search of three databases (PubMed, Embase, and the Cochrane Library) was conducted from their inception to May 2020 using the following keywords

(“miscarriage surgery”) and (“Prophylactic antibiotics”) and (“randomized controlled trial”). In addition, the references of relevant articles were hand-searched for records that may have been missed. The study selection procedure is presented in a PRISMA flow chart (Fig. 1).

### 2.2 Criteria for inclusion and exclusion

The eligible studies need to conform to the following inclusion criteria:

(1) randomized control trials (RCTs) enrolled patients undergoing miscarriage surgery;
(2) prophylactic antibiotics or placebo were used before surgery aiming at preventing the pelvic infection;
(3) if data were presented in more than 1 article, the most recent or the most elaborate study would be selected;
(4) reviews, case reports, editorial comments, or letters to the editor without original data were not included.

### 2.3 Outcomes of interest

Patient important outcomes for this study include pelvic infection within 14 days after miscarriage surgery, additional antibiotic use, additional analgesia (in addition to standard postoperative analgesia), unplanned hospital admissions, and adverse events included maternal death, diarrhea, vomiting, allergy, anaphylaxis, and blood transfusion.

### 2.4 Data extraction and quality assessment

Two investigators will independently extract data on the characteristics of the included studies (eg, first author name, publication year, intervention types, sample size), the outcome data (eg, events of infections, total sample size), and they will assess the risk of bias in individual studies by using the Cochrane Collaboration's Tool in the following aspects: The assessment includes sequence generation; allocation concealment; blinding of participants, personnel, and outcome assessors; incomplete outcome data; selective outcome reporting; and other sources of bias.^[23]^ Any differences between the authors on the data extraction and quality assessment will be resolved by discussion.

### 2.5 Statistical analysis

RevMan version 5.3 will be used to perform all calculations related to the meta-analysis. Dichotomous data will be calculated in terms of a fixed or random effect model and expressed by the relative risk with 95% confidence interval. Continuous data will be presented as mean difference and 95% confidence interval. The inconsistency index (*I*^2^) and the *χ*^2^-based Cochran Q statistic will be applied for heterogeneity detection between clinical trials. When assessing the difference in outcome, heterogeneity involving all trials will be examined. A value of *P* < .05 will be considered statistically significant.

### 2.6 Subgroup analysis

When there is obvious heterogeneity among included studies, we will perform a subgroup analysis in accordance with different study qualities, treatments, controls, and outcome measurements if possible.

### 2.7 Sensitivity analysis

In the case of sufficient trials data, the risk of bias tool will be used to assess methodological quality. If low-quality articles are deleted, a second meta-analysis will be performed. The results and effect size of the 2 meta-analyses will be compared and discussed.^[24]^

### 2.8 Reporting bias

When there are at least 10 included RCTs, we will conduct Funnel plot and Egger regression test to identify any possible reporting bias.^[25]^

### 2.9 Grading the quality of evidence

In this systematic review, the quality of evidence for the entire study is assessed using the “Grades of Recommendations Assessment, Development, and Evaluation” standard established by the World Health Organization and international organizations.^[16]^ To achieve transparency and simplification, the Grades of Recommendations Assessment, Development, and Evaluation system divides the quality of evidence into four levels: high, medium, low, and very low.

### 2.10 Ethics and dissemination

No individual patient data will be involved in this study; thus, no ethic approval is needed. We will publish this study at a peer-reviewed journal.

## 3 Discussion

A numerous RCTs have reported prophylactic antibiotics for preventing infections for patients undergoing miscarriage surgery. However, their results are still not consistent. Therefore, the purpose of this study is to determine the effectiveness and safety of prophylactic antibiotics in practice.

There are strengths in our study. First, this meta-analysis provides a comprehensive assessment to the benefits and harms of using prophylactic antibiotics for preventing infections for patients undergoing miscarriage surgery. Further, RCTs will be included in our studies and appear to be high quality and low risk of bias. However, there may be some limitations in our meta-analysis. This study may still have 2 limitations. First, some trials may have small sample size, which may affect results of this study. Second, the overall quality of some studies may be still low, which may impact study findings.

In conclusion, this study will help to determine the benefits and harms on the use of prophylactic antibiotics for preventing infections for women undergoing miscarriage surgery.

## Author contributions

**Conceptualization:** FY, RRJ

**Data curation:** XXW, QMS, XJL

**Formal analysis:** XZW

**Funding acquisition:** YHZ

**Methodology:** YF, RRJ, XXW

**Project administration:** YHZ

**Resources:** YHZ, YF

**Software:** ZFT, XYS

**Supervision:** YHZ

**Writing – original draft:** FY, RRJ

**Writing – review & editing:** YHZ
